# Involvement of Antizyme Characterized from the Small Abalone *Haliotis diversicolor* in Gonadal Development

**DOI:** 10.1371/journal.pone.0135251

**Published:** 2015-08-27

**Authors:** Wei-Dong Li, Min Huang, Wen-Gang Lü, Xiao Chen, Ming-Hui Shen, Xiang-Min Li, Rong-Xia Wang, Cai-Huan Ke

**Affiliations:** 1 Hainan Academy of Ocean and Fisheries Sciences, Haikou, Hainan Province, China; 2 College of Oceanography and Environmental Science, Xiamen University, Xiamen, Fujian Province, China; 3 Guangxi Key Lab for Mangrove Conservation and Utilization, Guangxi Mangrove Research Center, Beihai, Guangxi Province, China; Georg August University of Göttingen, GERMANY

## Abstract

The small abalone *Haliotis diversicolor* is an economically important mollusk that is widely cultivated in Southern China. Gonad precocity may affect the aquaculture of small abalone. Polyamines, which are small cationic molecules essential for cellular proliferation, may affect gonadal development. Ornithine decarboxylase (ODC) and antizyme (AZ) are essential elements of a feedback circuit that regulates cellular polyamines. This paper presents the molecular cloning and characterization of AZ from small abalone. Sequence analysis showed that the cDNA sequence of *H*. *diversicolor* AZ (HdiODCAZ) consisted of two overlapping open reading frames (ORFs) and conformed to the +1 frameshift property of the frame. Thin Layer chromatography (TLC) analysis suggested that the expressed protein encoded by +1 ORF2 was the functional AZ that targets ODC to 26S proteasome degradation. The result demonstrated that the expression level of AZ was higher than that of ODC in the ovary of small abalone. In addition, the expression profiles of ODC and AZ at the different development stages of the ovary indicated that these two genes might be involved in the gonadal development of small abalone.

## Introduction

Abalone is a common name of a group of marine sea snail belonging to gastropod molluscs. In the evolutionary history, abalone appears earlier than mammals and lies between Coelomata and Euteleostomi. Abalone is an economically important marine shellfish that is increasingly utilized in aquaculture worldwide [1]. The small abalone *H*. *diversicolor* is distributed in the coast of South China and Japan. The aquaculture of *H*. *diversicolor* has recently encountered a series of problems, including gonad precocity and slow growth rate. The mechanism underlying the gonadal development of *H*. *diversicolor* at the cellular molecular levels is still unclear. Nevertheless, molecules such as gonadotropin-releasing hormone (GnRH) are reportedly involved in the gonadal development of higher animals [2, 3]. It was suggested that polyamines may also play an important role in gonadal development [4, 5].

Polyamines such as putrescine, cadaverine, spermidine, and spermine are small basic molecules that play an important role in fundamental cellular processes, including ion channel function, DNA folding, replication, transcription, and translation [4, 6, 7]. Previous studies demonstrated that polyamines have high concentrations in developing tissues and tumor cells [8]. All cells can synthesize polyamines, and most cells can absorb polyamines across the plasma membrane [9–12]. Intracellular polyamine levels are regulated and primarily depend on the activity of ornithine decarboxylase (ODC, EC 4.1.1.17). ODC is the first rate-limiting enzyme in the polyamine biosynthetic pathway; this enzyme catalyzes the synthesis of putrescine, which is further converted into the polyamines spermidine and spermine [12–16]. ODC has a very short half-life and is rapidly degraded by 26s protease when polyamine levels increase [17, 18].

ODC degradation is controlled by antizymes (AZs), a class of proteins that respond to polyamine concentration. AZs were originally described as ODC inhibitors [19]. The affinity of AZs toward ODC subunits is higher than that of ODC subunits to one another; hence, AZs can easily bind to transient ODC subunits to form inactive ODC/AZ heterodimers and target ODC to ubiquitin-independent proteasomal degradation [20–22]. It is suggested that interaction with AZs would expose C-terminal proteasome degradation signal of ODC [23]. AZs reduce cellular polyamine concentration not only by promoting ODC degradation but also by interfering with the polyamine transport of external polyamines via an unknown mechanism [24–26]. The capacities of AZs to degrade ODC, inhibit polyamine uptake, and consequently suppress cellular proliferation make AZs act as tumor suppressors. Maintaining the balance between ODC and AZ is apparently important for normal cell growth, because ODC overproduction was associated with neoplastic transformation [27, 28] and AZ overexpression was indicated to inhibit cell growth [29]. AZs are synthesized from two open reading frames (ORFs) via a unique polyamine-stimulated ribosomal frameshifting [30–32]. The functional part of AZs is encoded by a +1 open reading frame (i.e., ORF2); thus, ribosomes should be subverted to the +1 reading frame to be translated into mature functional antizymes. This frameshift is stimulated by a pseudoknot structure located at 3′ to the frameshift site in the AZ mRNA [33]. The frameshifting efficiency is affected by the levels of cellular polyamines. A high polyamine concentration is likely to increase frameshifting efficiency, promote AZ synthesis, increase ODC degradation rate, inhibit polyamine uptake, and thus reduce the polyamine concentration in the cell. Therefore, AZs can be regarded as a biosensor for intracellular free polyamines [33]. AZ genes have been identified from yeasts to mammalians [31, 32] but not in bacteria. The frameshift mechanism is conservative, and proteins possess conservative domains from yeasts to mammalians [34, 35].

In the present study, we isolated, expressed in vitro, and identified the function of *H*. *diversicolor* antizyme (HdiODCAZ) and investigated the effect of AZ on the cellular polyamine synthesis and cell proliferation in abalone. Analysis on the expression pattern of HdiODCAZ in different tissues and in different developmental stages were also performed.

## Materials and Methods

### 1. Amplifying and sequencing the complete sequence of HdiODCAZ

Following the procedure described by Li et al. [36], we isolated the total RNA of small abalone hemocyte by using a TRIzol kit and conducted reverse transcription polymerase chain reaction (RT-PCR) to generate cDNA clones. The design of the degenerate primers used to amplify the HdiODCAZ sequence was based on the published partial sequence of HdiODCAZ mRNA in the National Center for Biotechnology Information (NCBI) (accession number EU244375) [37]. The designed primers were AZ-up-1 (5′-ATCCCCTTCGTCAGAGTCTTCCT-3′) and AZ-up-2 (5′-GACGGAGAAGCCCAGGAAACTGA-3′). The amplified segments were inserted into the cloning vector pMD-18T and were transferred into competent cells of *E*. *coli* DH 5α. The positive clone was selected and sequenced. The primers for 5′ RACE and 3′ RACE were designed on the basis of the sequenced data, and segments from the 5′ and 3′ RACE of the first-strand cDNA solution were also cloned and sequenced.

### 2. Analysis of HdiODCAZ sequence

The ORFs in the complete mRNA sequence of HdiODCAZ were identified using an ORF finder (http://www.ncbi.nlm.nih.gov/projects/gorf/orfig.cgi), and then the nucleotide sequences were translated into amino acids using the Vector NTI 11 software. The AZ sequence was analyzed using the ODC AZ finder software [38], and the codon region and frameshifting site were identified.

Homology searches were performed using BLASTn and BLASTp in NCBI. The Conserved Domain (CD) Search service was used to identify the CDs in the predicted protein sequences (http://www.ncbi.nlm.nih.gov/Structure/cdd/cdd.shtml). 3-D structure of the predicted protein were predicted according to the methods described in the website http://bioinf.cs.ucl.ac.uk/psipred/. The deduced amino acid sequence of HdiODCAZ was aligned using the CLC main workbench software (http://www.clcbio.com) with the known homologous proteins of the AZ class obtained from GenBank. A phylogenetic tree was reconstructed using the CLC main workbench software through the neighbor-joining method for the amino acid sequences of AZs from the SwissProt databank/Genbank: red jungle fowl, *Gallus gallus* (O42148); human, *Homo sapiens* (P11926); house mouse, *Mus musculus* (P54369); Norway rat, *Rattus norvegicus* (P09057); pig, *Sus scrofa* (NP_001116466); cattle, *Bos taurus* (P27117); fruit fly, *Drosophila melanogaster* (P54361); mosquito, *Aedes aegypti* (Q95P51); red flour beetle, *Tribolium castaneum* (NP_001242998); yeast, *Schizosaccharomyces pombe* (Q9USQ5); sea purse, *Triplophysa marmorata* (AAG16236); zebrafish, *Danio rerio* (Q9YI97, Q9YI98); Atlantic salmon, *Salmo salar* (NP_001134904); African clawed frog, *Xenopus laevis* (P55814); zebra finch, *Taeniopygia guttata* (NP_001166235); and small abalone (ACV32415).

### 3. Protein expression and activity determination of HdiODCAZ

HdiODCAZ was expressed in *Escherichia coli* HT414 as described by Li et al. [36]. Two ORFs were identified in the AZ gene; thus, two proteins called AZORF1 and AZ+1ORF2 were expressed. The coding regions were subcloned into the expression vector pGEX-4T-2, which could be expressed as a recombinant protein with a C-terminal fusion glutathione S-transferase tag. The expressed proteins were confirmed through 12% sodium dodecyl sulfate–polyacrylamide gel electrophoresis (SDS–PAGE) as described by Laemmli [39]. The enzyme activity of HdiODCAZ was identified as described by Gandre et al. [40] using a 26s proteinase degeneration kit. The different treatments were as follows: 1) the supernatant of 4T-2 reacted with ornithine; 2) the supernatant of ODC reacted with ornithine; 3) the supernatant of ODC and the inhibitor difluoromethylornithine (DFMO) reacted with ornithine; 4) the supernatants of ODC and AZORF1 reacted with ornithine; 5) the supernatants of ODC and AZ+1ORF2 reacted with ornithine; 6) the supernatants of ODC and 26s protease reacted with ornithine; 7) the supernatant of ODC and the supernatants of AZORF1 and 26s protease reacted with ornithine; and 8) the supernatant of ODC and the supernatants of AZ+1ORF2 and 26s protease reacted with ornithine. The reaction products were identified through thin-layer chromatography (TLC) as described by García–Moruno et al. (2005) and De las Rivas et al. (2008) [41, 42].

### 4. Expression of AZ in different tissues

The tissue distribution of AZ in small abalone was examined in various organs, including the muscles, digestive gland, hemocyte, tentacle, mantle, gill, and ovary. The total RNAs from various tissues were extracted using a TRIzol reagent. The expression of AZ in the different tissues was identified through quantitative real-time PCR (Invitrogen, USA) with HdiODCAZ specific primers AZ-up-1 and AZ-up-2. Actin was used as a reference gene to normalize the amount and quality of each cDNA for the reason that this gene were expressed constitutively in different tissues [43, 44].

### 5. Expression of AZ at different gonadal development stages

The gonadal development was divided into five stages based on the covering areas on the digestive gland. Tissue cells from the small abalone ovaries at four development stages except the resting stage, were separated to determine the expression of AZ. Two samples with three replicates were used for each stage. The total RNAs were extracted from the ovaries at different development stages using a TRIzol reagent. The expression levels of ODC and AZ were determined via quantitative real-time PCR with HdiODCAZ specific primers AZ-up-1 and AZ-up-2. Statistical analysis was conducted by SPSS 19.0 (IBM Corporation) and T-test was performed to test the statistical significance for the different expression.

## Results

### 1. Analysis of HdiODCAZ cDNA sequence and deducible amino acid sequences

The cDNA sequence of HdiODCAZ was cloned and submitted to Genbank with the accession number FJ809756.

**Fig 1:**
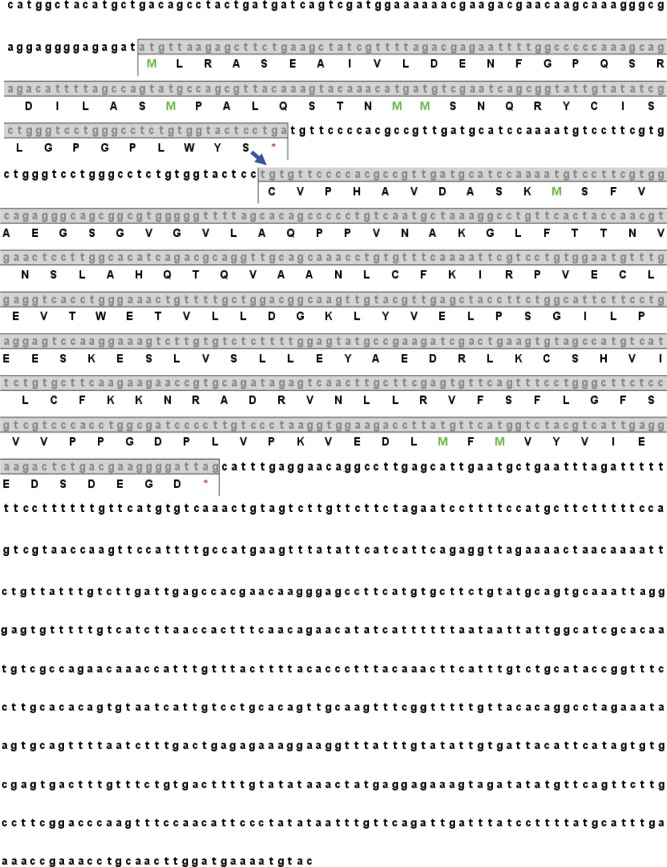
Sequences of small abalone antizyme nucleic acid and deducible amino acids.

The nucleotide sequence and the predicted amino acid sequence of HdiODCAZ are presented in **Fig 1**. HdiODCAZ nucleic acids were 1434 bp long. The red asterisk indicated the first stop codon TGA, and the small ORF (ORF1) coded for a nonfunctional protein that was composed of 51 amino acids. Different from the usual two start codons in most of higher vertebrates, four start codons (ATG) were found in ORF1 of HdiODCAZ [40]. Many previous studies predicted the frameshifting properties in AZ sequences, and the analysis of HdiODCAZ cDNA sequence in our study confirmed this conclusion. In this case, the ribosome skipped nucleotide A in the stop codon TGA and read TGT instead during the translation. Thus, frameshift occurred, and +1 ORF2 encoded for a functional AZ composed of 212 amino acids. The conserved sequence TCCTGATGT existed around the frameshift site. The 5′ untranslated region (UTR) of the cDNA sequence was very short, whereas the 3′ UTR was remarkably long. The typical polyA recognition site (AATAAA) was absent in the 3′ UTR, but this site was substituted by TATAAA and AAATAA.

### 2. Analysis of deducible proteins

**Fig 2:**
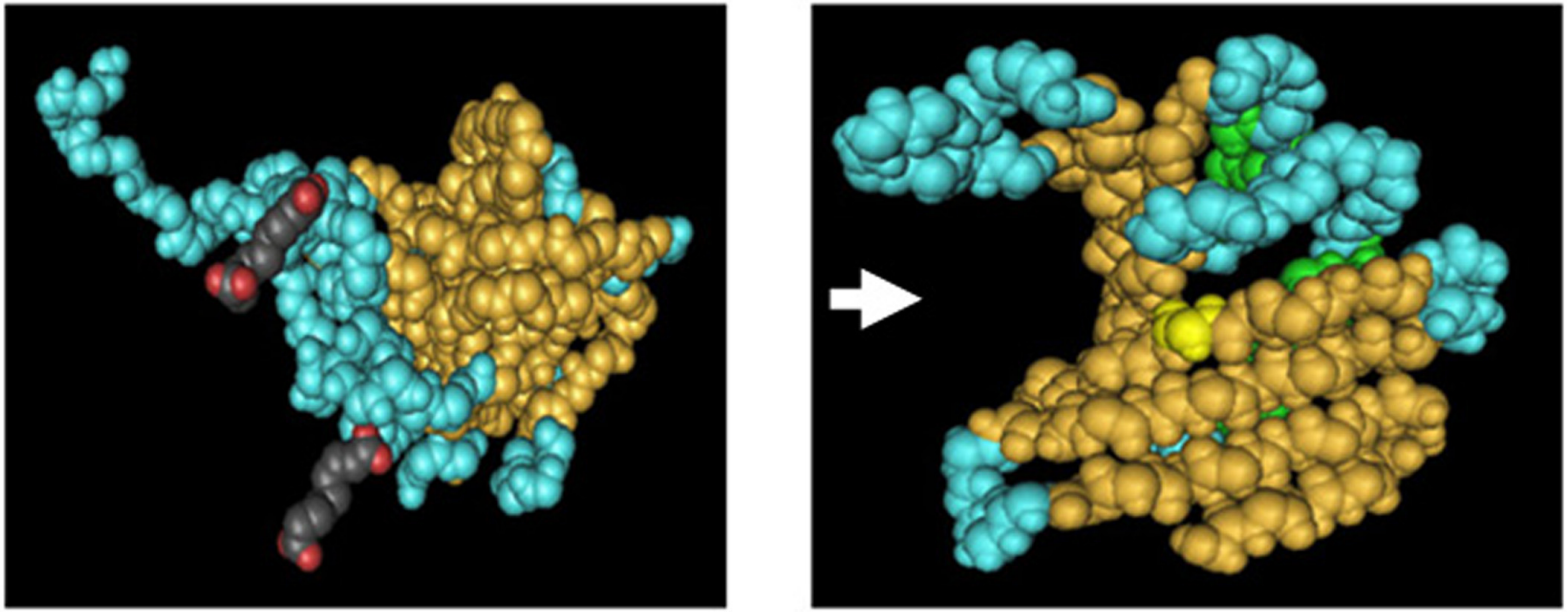
C-terminal 3-D structure comparison of small abalone AZ and ODC (A) C-terminal 3D structure of ODC (B) C-terminal 3D structure of AZ.

As showed in **Fig 2**, the C-terminal 3D structure of abalone AZ appears as a clamp. This structure allows AZ to fuse to ODC and form a heterodimer.

**Fig 3:**
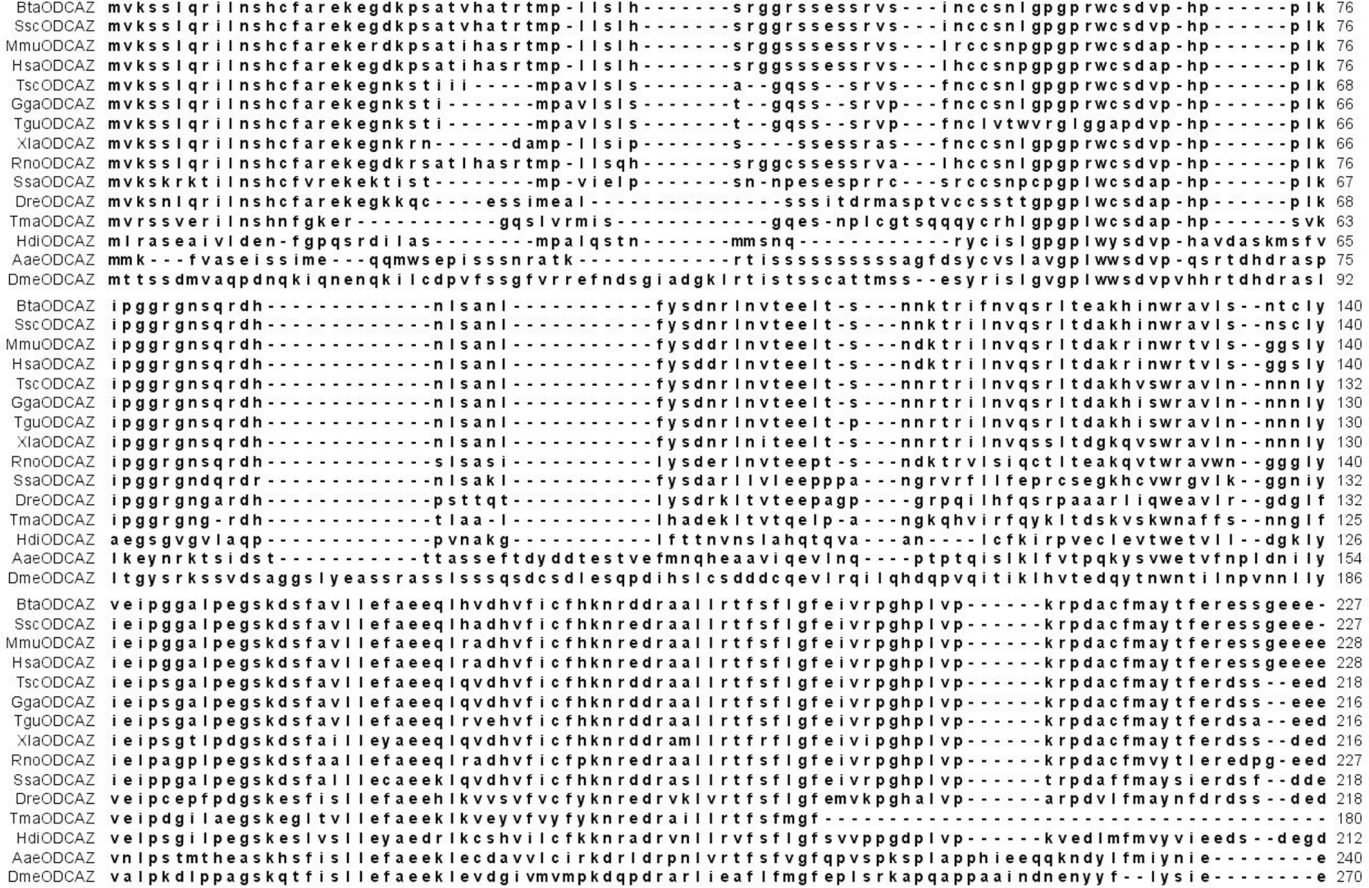
Multiple alignment of the predicted AZ amino acid sequence with known AZ protein sequences.

The predicted amino acid sequence of HdiODCAZ was aligned with the known AZ sequences through BLASTp. As shown in **Fig 3**, the small abalone AZ protein sequence shared a low percentage of similarity to other known AZ protein sequences. This result indicates that members of the AZ family are more diverged from one another than those of the ODC family. The amino acid sequence of HdiODCAZ was approximately 44% identical to those of AZs from Norway rat and house mouse, approximately 43% identical to those of AZs from red jungle fowl and zebra finch, and 41% identical to that of AZ from humans. The highest level of similarity appeared near to the C-terminal, and the similarity in the N-terminal and in the middle of the amino acid sequence was very low.

**Fig 4:**
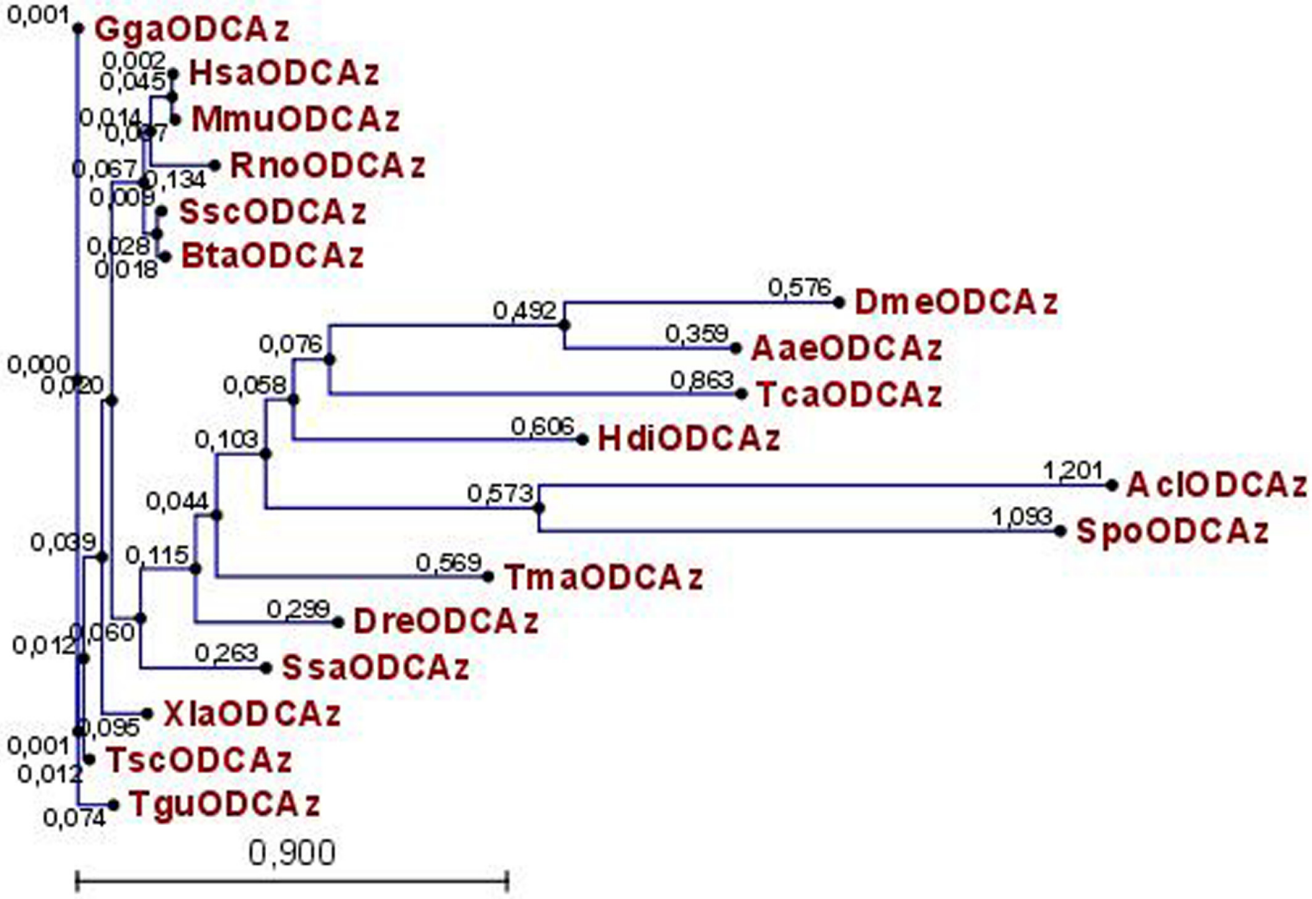
A phylogenetic tree of small abalone antizyme with known AZ. The tree is constructed by the neighbor-joining method based on an alignment corresponding to full-length amino acid sequences, using CLC main workbench. The length of the tree branches indicated the evolutionary relation of those species.

A molecular phylogenetic tree was constructed to analyze the evolutionary relationship of AZ amino acid sequences (**Fig 4**). The tree showed that the AZ of small abalone evolutionally shared a higher sequence identity with fruit fly, mosquito, zebra fish, and sea purse. These results indicate that this gene may be used as an evolutionary marker.

**Fig 5:**
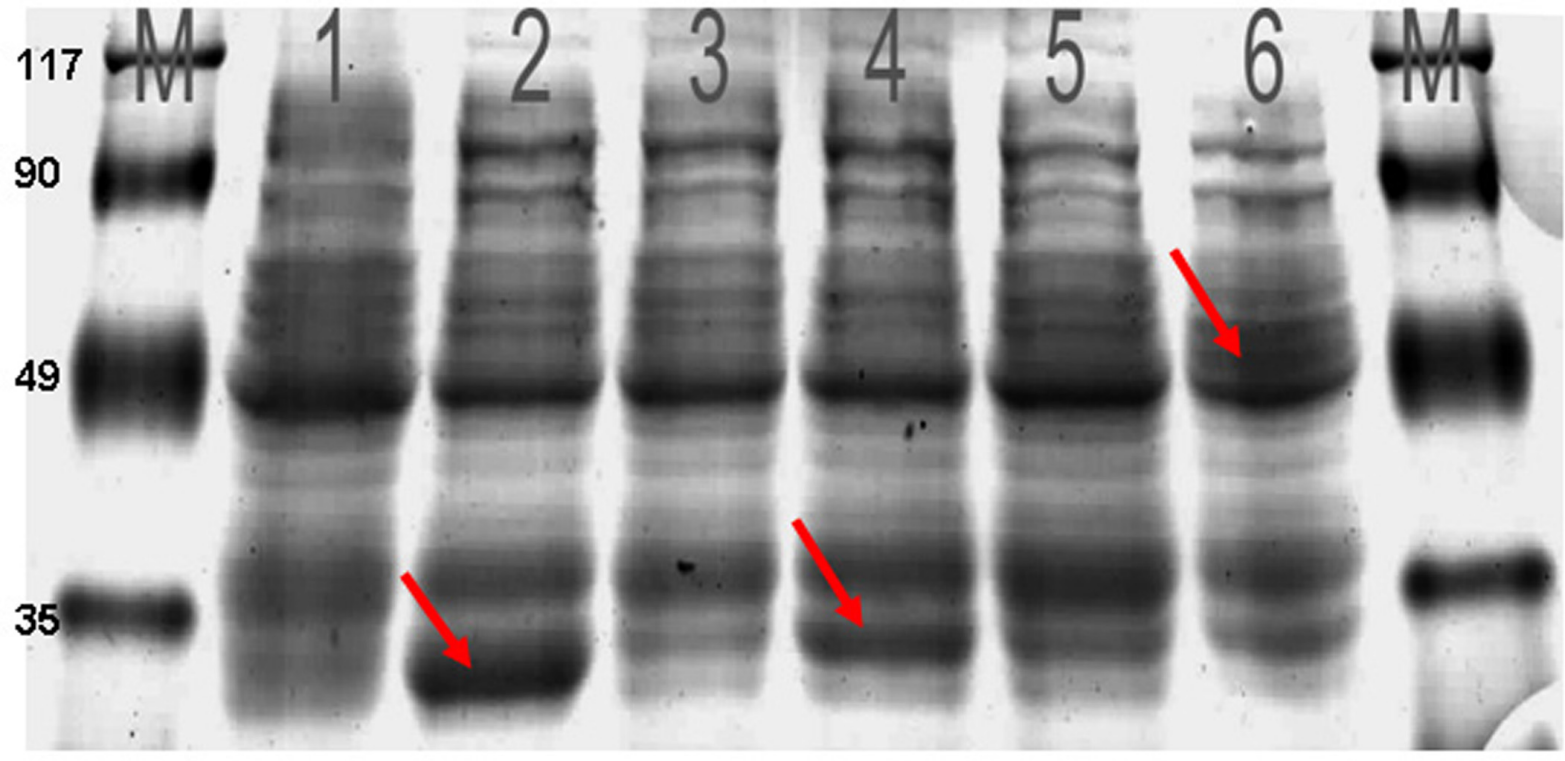
SDS-PAGE analysis of Az ORFs overexpression by E. coli HT414 strain. (M) Molecular weight marker (1–6) Electrophoresis separations of cytosolic proteins from E. coli HT414 (1) The solution from E. coli HT414 that contained the plasmid pGEX-4T-2 (2) induced solution of IPTG from E. coli HT414 that contained the plasmid pGEX-4T-2 plasmid and produced the tag-protein GST (red arrowhead point) (3) the solution from E. coli HT414, which contained the plasmid pGEX-4T-2-AzORF1 (4) induced solution of IPTG from E. coli HT414, which contained the plasmid pGEX-4T-2-AZORF1 and produced the fused protein GST-AZORF1 (red arrowhead point) (5) The solution from E. coli HT414, which contained the plasmid pGEX-4T-2-AZ+1ORF2 (6) induced solution of IPTG from E. coli HT414, which contained the plasmid pGEX-4T-2-AZ+1ORF2 and produced the fusion protein GST-AZ+1ORF2 (red arrowhead point).

SDS-PAGE analysis (**Fig 5**) indicated that the fusion protein GST-AZORF1 was approximately 32 kDa, whereas the fusion protein GST-AZ+1ORF2 was approximately 50 kDa.

**Fig 6:**
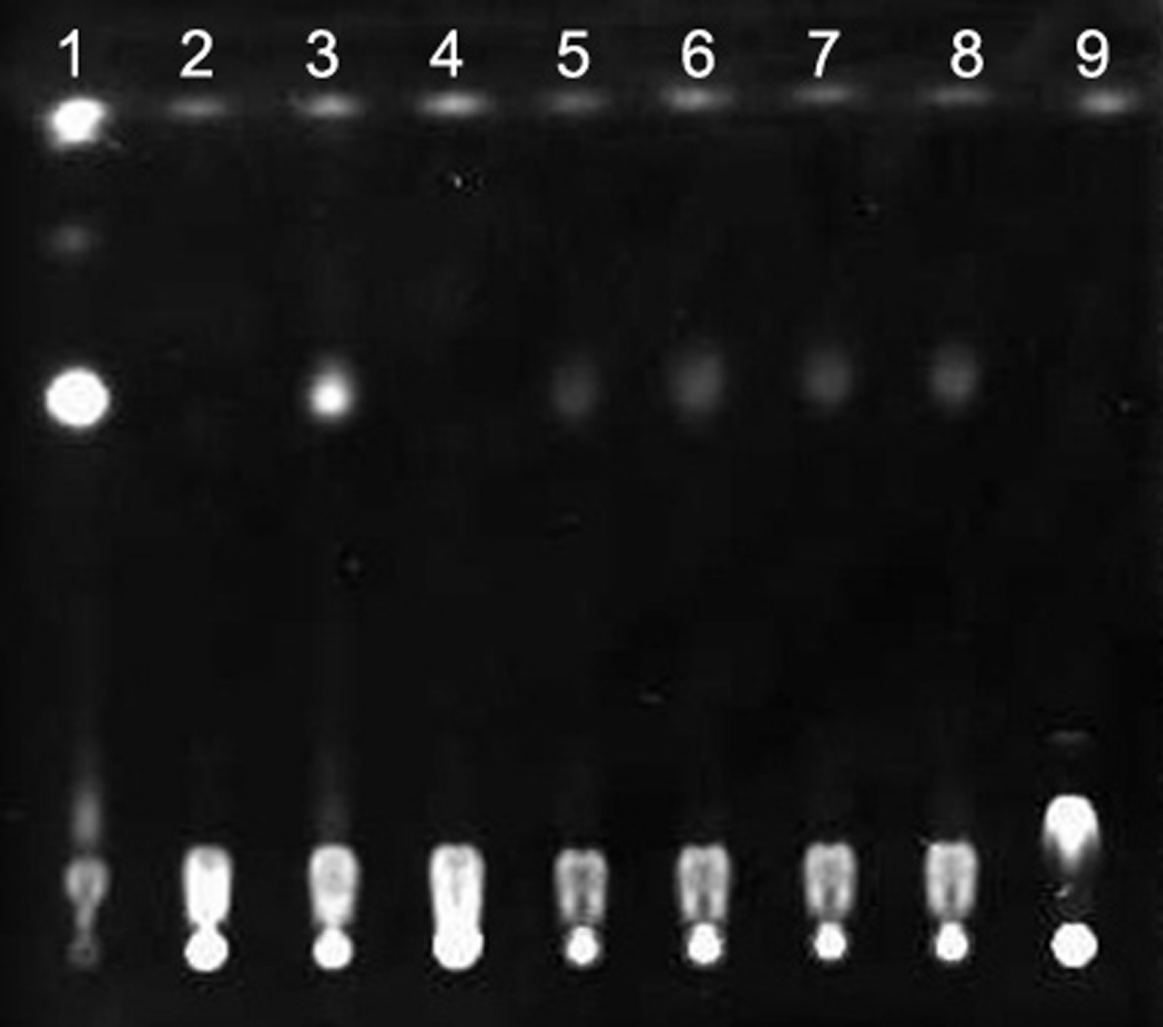
TLC analysis of overexpressed proteins of Az ORFs reaction to L-ornithine. The putrescine produced during the enzymatic reactions was separated on a precoated silica gel 60 F254 plate (Merck, Darmstadt, Germany). (1) Control putrescine standard solution (2) Putrescine was not produced, in the supernatant from E. coli HT414 that contained plasmid pGEX-4T-2 and generated the tag-protein GST, reacted with substrate L-ornithine (3) Putrescine was produced, in the supernatant from E.coli HT414 that contained the plasmid pGEX-4T-2-ODC and generated the recombinant protein GST-ODC, reacted with L-ornithine (4) Putrescine was not produced, in the supernatant from E. coli HT414 that contained the plasmid pGEX-4T-2-ODC and the inhibitor DFMO, reacted with L-ornithine (5) Putrescine was produced, in the supernatant from E.coli HT414 that contained the plasmid pGEX-4T-2-ODC and the plasmid pGEX-4T-2-AZ ORF1, and generated the recombinant proteins GST-ODC and GST-AZ ORF1, reacted with L-ornithine (6) Putrescine was produced, in the supernatant from E. coli HT414 that contained the plasmid pGEX-4T-2-ODC and the plasmid pGEX-4T-2-AZ+1 ORF2, and generated the recombinant proteins GST-ODC and GST-AZ+1 ORF2, reacted with L-ornithine (7) Putrescine was produced, in the supernatant from E. coli HT414 that contained the plasmid pGEX-4T-2-ODC and generated the recombinant protein GST-ODC, with 26s protease and ATP existed, reacted with L-ornithine (8) Putrescine was produced, in the supernatant from E. coli HT414 that contained the plasmid pGEX-4T-2-ODC and pGEX-4T-2-AZ ORF1 and generated the recombinant proteins GST-ODC and GST-AZ ORF1, reacted with L-ornithine, with 26S protease and ATP existed (9) Putrescine was not produced, in the supernatant from E. coli HT414 that contained the plasmid pGEX-4T-2-ODC and pGEX-4T-2-AZ+1 ORF2 and generated the recombinant proteins GST-ODC and GST-AZ +1 ORF2, reacted with L-ornithine, with 26S protease and ATP existed

TLC analysis of the overexpressed proteins proved that the cloned nucleic acid fragments were abalone mRNAs coding for AZ. As shown in **Fig 6**, ODC catalyzed L-ornithine to generate putrescine, and the activity of ODC was inhibited by DFMO. The overexpressed protein of AZORF1, AZ+1ORF2, or 26s protease did not prevent putrescine production catalyzed by ODC individually. The overexpressed protein of AZORF1 did not inhibit putrescine generation with the existence of 26S protease and ATP. However, the overexpressed protein of AZ+1ORF2 inhibited putrescine generation. These results demonstrated that the overexpressed protein of abalone AZORF1 was the nonfunctional AZ, whereas the expressed protein of AZ+1ORF2 was the functional AZ that promoted ODC degradation through 26S protease.

### 3. Expression distribution of ODC and AZ in abalone

#### 3.1 AZ expression in different tissues

**Fig 7:**
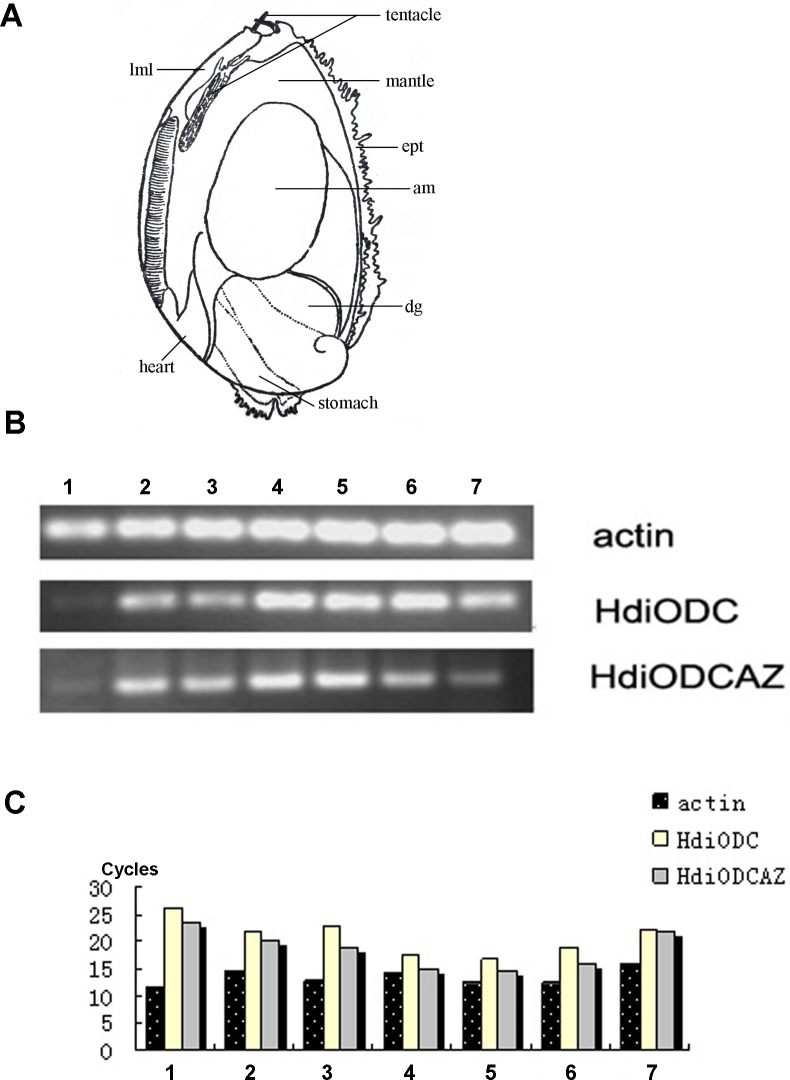
The tissue expression of HdiODCAz in small abalone, *H*. *diversicolor*. (A) Schematic representation of a de-shelled 5-mm-long juvenile of small abalone. The gills, heart, stomach, mantle, tentacles, digestive gland (dg), adductor muscle (am), epipodial tentacles (ept) and left mantle lobe (lml) are indicated. (B) The electrophoresis result of real-time PCR. (C) The pillar figure of real-time PCR. Actin gene is the control gene. Seven different tissues were chose to investigate the tissue expression pattern of AZ. (1) digestive gland (2) muscle (3) hemocyte (4) tentacle (5) mantle (6) gill (7) ovary.

The tissue distribution of the small abalone AZ gene was investigated through real time RT-PCR, with the total RNA isolated from small abalone tissue used as a template. As shown in Fig 7, the AZ gene was expressed at different levels in tissues from the muscle, digestive gland, hemocyte, tentacle, mantle, gill, and ovary of abalone. The highest expression quantity relative to actin was observed in the tentacle, which preceded the mantle and gill. By contrast, the lowest expression level was in the digestive gland, followed by the hemocyte, muscle, and ovary. The expression quantity of AZ was consistently slightly higher than that of ODC in almost all the tested tissues, except in the ovary. The expression quantities of AZ and ODC showed no significant difference in small abalone ovary.

#### 3.2. AZ expression at different gonadal development stages

**Fig 8:**
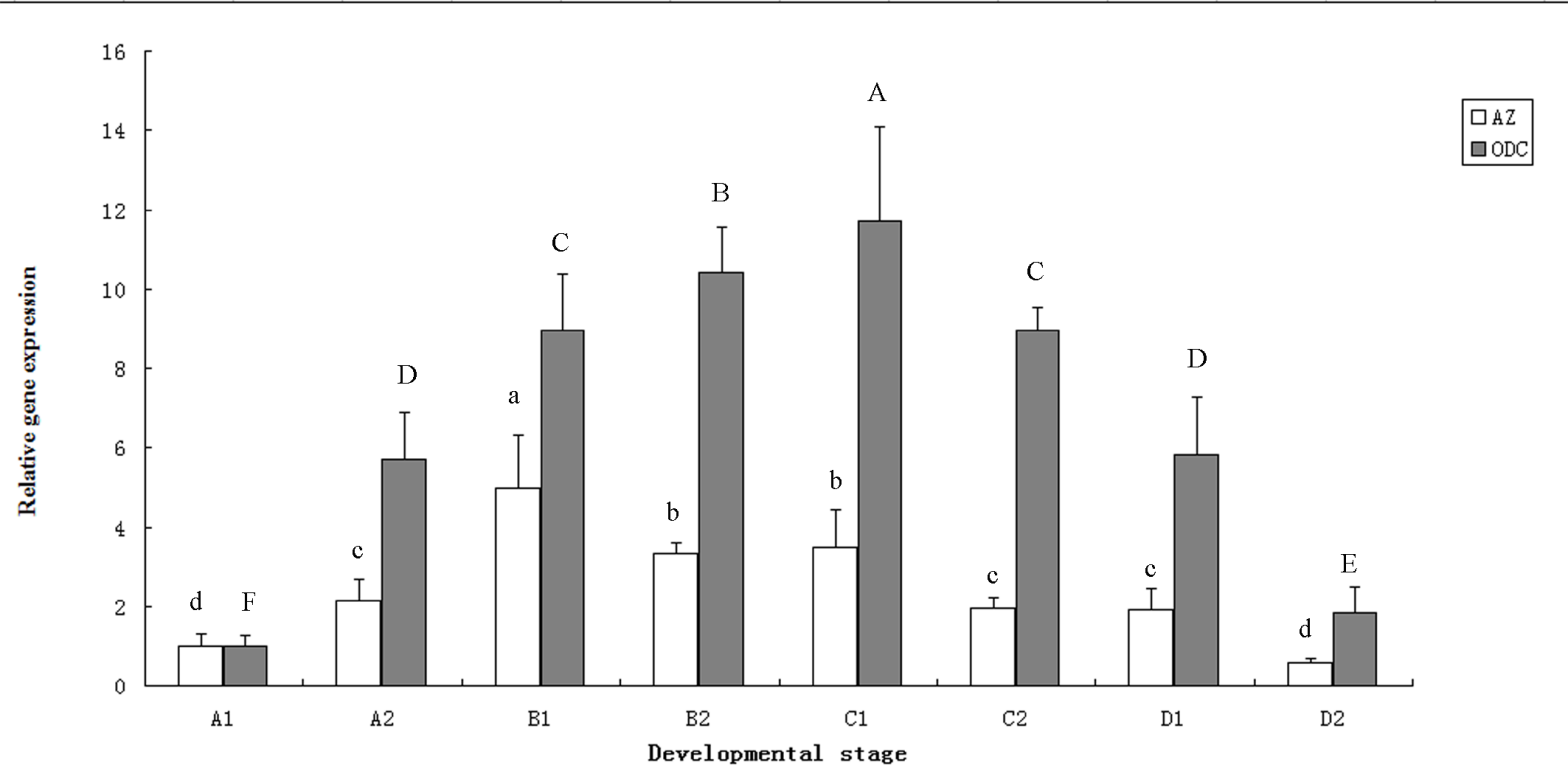
The expression of ODC and AZ in different ovary development stages. (A1)(A2) ovaries in proliferating stage (B1)(B2) ovaries in growth stage (C1)(C2) ovaries in maturing and spawning stage (D1)(D2) ovaries in final stage. The expression levels of ODC and AZ in A1 were set to 1, and the ratios of expression quantities in other groups to that in A1 were showed respectively. The letters in the figure indicated the significant difference from t-test (P<0.01). And the error bar represented the standard deviation.

The expression levels of ODC and AZ at the different gonadal development stages in the female small abalone were investigated. As shown in **Fig 8**, the expression of ODC at the four developmental stages initially increased, decreased, and then peaked at the maturing and spawning stages (C1). The final expression of ODC was more than 10 times higher than the initial expression. The expression quantity at the final stage was almost the same as that at the initial stage. Similar to the expression of ODC, that of AZ also increased and then decreased. However, the peak expression of AZ was detected at the growth stage (B1). The final expression of AZ was approximately five times higher than the initial expression.

## Discussion

Antizymes affect cell proliferation and viability by regulating cellular polyamines [45]. The AZ approach to deplete cellular polyamine concentration has been investigated by many researchers. The expressed AZ protein would significantly reduce ODC activity, but AZ overexpression can only slightly influence cellular polyamine concentration [46]. This result indicates that AZs regulate cell proliferation by targeting proteins that do not belong to the cellular polyamine metabolic pathway. Research [47] suggested that AZ regulated intestinal cell growth independent of polyamines. AZ also facilitates the degradation of a set of regulatory proteins, including Cyclin D1 [48], SMAD1 [49], Aurora-A kinase [50], and Mps1 [51, 52]. Research on ODC AZ from abalone is limited. The AZ gene was validated to be used as a single nucleotide polymorphism marker to evaluate the loss of genetic diversity due to hatchery selection [53] or internal control gene [54] in abalone.

This study is the first to isolate, sequence, and characterize cDNA clones that encode AZs from small abalone. Two overlapping ORFs in the HdiODCAZ sequence illustrated the frameshifting property of the sequence; this finding suggested that HdiODCAZ was a member of the AZ family (**Fig 1**). Numerous studies demonstrated that the AZ family has at least four members. AZ 1 promotes ODC degradation through 26S proteasome, inhibits polyamine uptake, and stimulates polyamine excretion [55]. AZ 2 is less abundantly expressed than AZ 1 [56] and does not promote ODC degradation [57]. Research also suggested that AZ 2 can augment the effects of AZ 1 on the follicle development of Sichuan white goose [58]. The expression of AZ 3 is restricted to certain stages of spermatogenesis [59]. Lastly, AZ 4 was originally isolated from a human brain cDNA library [32], and the function of this AZ family remains uncharacterized. The isolation and characterization of the other AZ members in small abalone require further research.

AZ may be expressed in prokaryotic expression systems for the reason that currently there is no AZ gene found in prokaryotes. Functional determination indicated that the expressed proteins of HdiODCAZ did not work directly but regulated ODC degradation. Previous research demonstrated that AZ and ODC would form an ODC/AZ dimer that facilitates ODC degradation through 26S proteasome. It was shown in **Fig 6**that the expressed protein of AZORF1 is nonfunctional, whereas that of AZ+1ORF2 is functional and can augment ODC degradation.

AZ is not a tissue-specific gene and can be expressed in various tissues from different small abalone organs. The expression distribution of AZ is in accordance with that of ODC but with slight differences (**Fig 7**), suggested that the expression levels of AZ and ODC may reach a variable balance. This finding confirms the correlation of AZ and ODC, implying that AZ may be used as a negative regulator of ODC. The lowest expression quantity of AZ relative to ODC was in the ovary; thus, the highest ODC expression was detected in the ovary of small abalone.

According to the size of the areas or the extent of the covering on the digestive gland, the gonadal development in female small abalone is commonly divided into five stages: resting, proliferating, growing, maturing and spawning, and final stage. The expression levels of ODC and AZ at the four development stages of the ovary increased and then decreased (**Fig 8**). This result agrees with the growth curve of the germs in the ovary. In general, almost no gonadal tissue appears at the resting stage which starts on January; at the proliferating stage from February to April germ cells start to appear and the gonad covers about one fourth of digestive gland; germ cells rapidly reproduce and the gonad thickens at the growing stage from May to July, and the gonad would cover approximate half to two third of digestive gland; germ cells become mature and start to spawn at the maturing and spawning stages from August to October, the digestive gland is fully covered by the gonad; and the gonad shrink at the final stage from November to next January (**S1 Fig**). The highest expression level of ODC was detected at the maturing and spawning stage (C1) because the fully developed ovary at this stage produces large amounts of gametes and requires a high concentration of cellular polyamines. The expression of AZ at the different stages would balance the expression levels of ODC because AZ is a negative ODC regulator. The results demonstrate that ODC and AZ significantly affect the development of small abalone gonad. To prevent gonad precocity of small abalone further research for details is required.

## Supporting Information

S1 Fig Schematic representation of the different gonadal development stages in small abalone. The digestive system of small abalone were shown. The gray area indicated the gonad tissues. The gonadal development were divided into five stages based on the size of gonad tissues or the extent of the covering on the digestive gland. (1) resting stage (2) proliferating stage (3) growth stage (4) maturing and spawning stage (5) final stage.
(TIF)

## References

[1] Cook P, Gordon HR. World abalone supply, markets and pricing. J Shellfish Res. 2010, 29(3): 569–71.

[2] Metallinou C, Asimakopoulos B, Schröer A, Nikolettos N. Gonadotropin-releasing hormone in the ovary. Reprod Sci. 2007; 14(8): 737–49. 18089592 10.1177/1933719107310707

[3] Nuurai P, Poljaroen J, Tinikul Y, Cummins S, Sretarugsa P, Hanna P, et al The existence of gonadotropin-releasing hormone-like peptides in the neural ganglia and ovary of the abalone, *Haliotis asinina* L. Acta Histochem. 2010; 112(6): 557–66. 10.1016/j.acthis.2009.06.002 19604545

[4] Cohen SS. A guide to the polyamines New York: Oxford University Press; 1998 pp. 231–259.

[5] Liu L, Guo X, Rao JN, Zou T, Xiao L, Yu T, et al Polyamines regulate E-cadherin transcription through c-Myc modulating intestinal epithelial barrier function. Am J Physiol Cell Physiol. 2009; 296(4): C801–10. 10.1152/ajpcell.00620.2008 19176757 PMC2670658

[6] Bardócz S, White A. Polyamines in health and nutrition Norwell, MA: Kluwer Academic Publishers; 1999 pp. 1–77.

[7] Shantz LM, Pegg AE. Translational regulation of ornithine decarboxylase and other enzymes of the polyamine pathway. Int J Biochem Cell Biol. 1999;31: 107–22. 10216947 10.1016/s1357-2725(98)00135-6

[8] Schipper RG, Romijn JC, Cuijpers VM, Verhofstad AA. Polyamines and prostatic cancer. Biochem Soc Trans. 2003;31(2): 375–80. 12653642 10.1042/bst0310375

[9] Tabor CW, Tabor H. Polyamines. Annu Rev Biochem. 1984;53: 749–90. 6206782 10.1146/annurev.bi.53.070184.003533

[10] Pegg AE. Polyamine metabolism and its importance in neoplastic growth and a target for chemotherapy. Cancer Res. 1988;48(4): 759–74. 3123052

[11] Gerner EW, Meyskens FL. Polyamines and cancer: old molecules, new understanding. Nat Rev Cancer. 2004;4(10): 781–92. 15510159 10.1038/nrc1454

[12] Ueda A, Araie M, Kubota S. Polyamine depletion induces G1 and S phase arrest in human retinoblastoma Y79 cells. Cancer Cell Int. 2008;8: 2 10.1186/1475-2867-8-2 18208615 PMC2259317

[13] Russell DH. Ornithine decarboxylase as a biological and pharmacological tool. Pharmacology. 1980;20(3): 117–29. 6247722 10.1159/000137355

[14] Heby O, Persson L. Molecular genetics of polyamine synthesis in eukaryotic cells. Trends Biochem Sci. 1990;15(4): 153–8. 2187296 10.1016/0968-0004(90)90216-x

[15] Pegg AE. Regulation of ornithine decarboxylase. J Biol Chem. 2006;281(21): 14529–32. 16459331 10.1074/jbc.R500031200

[16] Müller IB, Das Gupta R, Lüersen K, Wrenger C, Walter RD. Assessing the polyamine metabolism of Plasmodium falciparum as chemotherapeutic target. Mol Biochem Parasitol. 2008;160(1): 1–7. 10.1016/j.molbiopara.2008.03.008 18455248

[17] Russell DH, Snyder SH. Amine synthesis in regenerating rat liver: extremely rapid turnover of ornithine decarboxylase. Mol Pharmacol. 1969;5(3): 253–62. 5783961

[18] Kay JE, Lindsay VJ. Control of ornithine decarboxylase activity in stimulated human lymphocytes by putrescine and spermidine. Biochem J. 1973;132(4): 791–6. 4721611 10.1042/bj1320791 PMC1177653

[19] Fong WF, Heller JS, Canellakis ES. The appearance of an ornithine decarboxylase inhibitory protein upon the addition of putrescine to cell cultures. Biochim Biophys Acta. 1976;428(2): 456–65. 179575 10.1016/0304-4165(76)90054-4

[20] Murakami Y, Matsufuji S, Kameji T, Hayashi S, Igarashi K, Tamura T, et al Ornithine decarboxylaseis is degraded by the 26S proteasome without ubiquitination. Nature. 1992;360(6404): 597–9. 1334232 10.1038/360597a0

[21] Hayashi S, Murakami Y, Matsufuji S. Ornithine decarboxylase antizyme: a novel type of regulatory protein. Trends Biochem Sci. 1996;21(1): 27–30. 8848835

[22] Coffino P. Regulation of cellular polyamines by antizyme. Nat Rev Mol Cell Biol. 2001;2(3): 188–94. 11265248 10.1038/35056508

[23] Li X, Coffino P. Degradation of ornithine decarboxylase: exposure of the C-terminal target by a polyamine-inducible inhibitory protein. Mol Cell Biol. 1993;13(4): 2377–83. 8455617 10.1128/mcb.13.4.2377-2383.1993 PMC359558

[24] Hoshino K, Momiyama E, Yoshida K, Nishimura K, Sakai S, Toida T, et al Polyamine transport by mammalian cells and mitochondria: role of antizyme and glycosaminoglycans. J Biol Chem. 2005;280(52): 42801–8. 16263714 10.1074/jbc.M505445200

[25] Mitchell JL, Judd GG, Bareyal-Leyser A, Ling SY. Feedback repression of polyamine transport is mediated by antizyme in mammalian tissue-culture cells. Biochem J. 1994;299 (Pt 1): 19–22. 8166639 10.1042/bj2990019 PMC1138014

[26] Suzuki T, He Y, Kashiwagi K, Murakami Y, Hayashi S, Igarashi K. Antizyme protects against abnormal accumulation and toxicity of polyamines in ornithine decarboxylase-overproducing cells. Proc Natl Acad Sci USA. 1994;91(19): 8930–4. 8090747 10.1073/pnas.91.19.8930 PMC44720

[27] Holtta E, Auvinen M, Andersson LC. Polyamines are essential for cell transformation by pp60v-src: Delineation of molecular events relevant for the transformed phenotype. J Cell Biol. 1993;122(4): 903–14. 7688751 10.1083/jcb.122.4.903 PMC2119593

[28] Shantz LM, Pegg AE. Ornithine decarboxylase induction in transformation by H-Ras and RhoA. Cancer Res. 1998; 58(13): 2748–53. 9661886

[29] Fong LY, Feith DJ, Pegg AE. Antizyme overexpression in transgenic mice reduces cell proliferation, increases apoptosis, and reduces N-nitrosomethylbenzylamine-induced forestomach carcinogenesis. Cancer res. 2003;63(14): 3945–54. 12873989

[30] Rom E, Kahana C. Polyamines regulate the expression of ornithine decarboxylase antizyme in vitro by inducing ribosomal frame-shifting. Proc Natl Acad Sci USA. 1994;91(9): 3959–63. 8171019 10.1073/pnas.91.9.3959 PMC43702

[31] Matsufuji S, Matsufuji T, Miyazaki Y, Murakami Y, Atkins JF, Gesteland RF, et al Autoregulatory frameshifting in decoding mammalian ornithine decarboxylase antizyme. Cell. 1995;80(1): 51–60. 7813017 10.1016/0092-8674(95)90450-6 PMC7133313

[32] Ivanov IP, Gesteland RF, Atkins JF. Antizyme expression: a subversion of triplet decoding, which is remarkably conserved by evolution, is a sensor for an autoregulatory circuit. Nucleic Acids Res. 2000;28(17): 3185–96. 10954585 10.1093/nar/28.17.3185 PMC110703

[33] Ivanov IP, Anderson CB, Gesteland RF, Atkins JF. Identification of a new antizyme mRNA +1 frameshifting stimulatory pseudoknot in a subset of diverse invertebrates and its apparent absence in intermediate species. J Mol Biol. 2004;339(3): 495–504. 15147837 10.1016/j.jmb.2004.03.082 PMC7125782

[34] Ivanov IP, Gesteland RF, Matsufuji S, Atkins JF. Programmed frameshifting in the synthesis of mammalian antizyme is +1 in mammals, predominantly +1 in fission yeast, but -2 in budding yeast. RNA. 1998;4(10): 1230–8. 9769097 10.1017/s1355838298980864 PMC1369695

[35] Ivanov IP, Atkins JF. Ribosomal frameshifting in decoding antizyme mRNAs from yeast and protists to humans: close to 300 cases reveal remarkable diversity despite underlying conservation. Nucleic Acids Res. 2007;35(6): 1842–58. 17332016 10.1093/nar/gkm035 PMC1874602

[36] Li W, You W, Chen W, Qin J, Huang Z, Ke C, et al Characterization of ornithine decarboxylase, a potential selective breeding marker, from small abalone, *Haliotis diversicolor* . J World Aquac Soc. 2010;41(5): 721–32.

[37] Wang KJ, Ren HL, Xu DD, Cai L, Yang M. Identification of the up-regulated expression genes in hemocytes of variously colored abalone (*Haliotis diversicolor* Reeve, 1846) challenged with bacteria. Dev Comp Immunol. 2008;32(11): 1326–47. 10.1016/j.dci.2008.04.007 18538840

[38] Bekaert M, Ivanov IP, Atkins JF, Baranov PV. Ornithine decarboxylase antizyme finder (OAF): Fast and reliable detection of antizymes with frameshifts in mRNAs. BMC Bioinformatics. 2008;9: 178 10.1186/1471-2105-9-178 18384676 PMC2375905

[39] Laemmli UK. Cleavage of structural proteins during the assembly of the head of bacteriophage T4. Nature. 1970;227(5259): 680–5 5432063 10.1038/227680a0

[40] Gandre S, Bercovich Z, Kahana C. Mitochondrial localization of antizyme is determined by context-dependent alternative utilization of two AUG initiation codons. Mitochondrion. 2003;2(4): 245–56. 16120325 10.1016/S1567-7249(02)00105-8

[41] García-Moruno E, Carrascosa AV, Muñoz R. A rapid and inexpensive method for the determination of biogenic amines from bacterial cultures by thin-layer chromatography. J Food Prot. 2005;68(3): 625–29. 15771195 10.4315/0362-028x-68.3.625

[42] De LasRivas B, González R, Landete JM, Muñoz R. Characterization of a second ornithine decarboxylase isolated from Morganella morganii. J Food Prot. 2008;71(3): 657–61. 18389719 10.4315/0362-028x-71.3.657

[43] Stürzenbaum SR, Kille P. Control genes in quantitative molecular biological techniques: the variability of invariance. Comp Biochem Physiol B Biochem Mol Biol. 2001;130(3): 281–9. 11567890 10.1016/s1096-4959(01)00440-7

[44] Li Z, Yang L, Wang J, Shi W, Pawar RA, Liu Y, et al beta-Actin is a useful internal control for tissue-specific gene expression studies using quantitative real-time PCR in the half-smooth tongue sole Cynoglossus semilaevis challenged with LPS or Vibrio anguillarum. Fish Shellfish Immunol. 2010;29(1): 89–93. 10.1016/j.fsi.2010.02.021 20227507

[45] Bercovich Z, Snapir Z, Keren-Paz A, Kahana C. Antizyme affects cell proliferation and viability solely through regulating cellular polyamines. J Biol Chem. 2011;286(39): 33778–83. 10.1074/jbc.M111.270637 21832059 PMC3190834

[46] Pietilä M, Dhungana H, Uimari A, Sironen R, Alhonen L. Systemic overexpression of antizyme 1 in mouse reduces ornithine decarboxylase activity without major changes in tissue polyamine homeostasis. Transgenic Res. 2014;23(1): 153–63. 10.1007/s11248-013-9763-y 24174210

[47] Ray RM, Bhattacharya S, Bavaria MN, Viar MJ, Johnson LR. Antizyme (AZ) regulates intestinal cell growth independent of polyamines. Amino Acids. 2014;46(9): 2231–9. 10.1007/s00726-014-1777-0 24930035 PMC4134379

[48] Newman RM, Mobascher A, Mangold U, Koike C, Diah S, Schmidt M, et al Antizyme targets cyclin D1 for degradation. A novel mechanism for cell growth repression. J Biol Chem. 2004;279(40): 41504–11. 15277517 10.1074/jbc.M407349200

[49] Gruendler C, Lin Y, Farley J, Wang T. Proteasomal degradation of Smad1 induced by bone morphogenetic proteins. J Biol Chem. 2001;276(49): 46533–43. 11571290 10.1074/jbc.M105500200

[50] Lim SK, Gopalan G. Antizyme1 mediates AURKAIP1-dependent degradation of Aurora-A. Oncogene. 2007;26(46): 6593–603. 17452972 10.1038/sj.onc.1210482

[51] Kasbek C, Yang CH, Fisk HA. Antizyme restrains centrosome amplification by regulating the accumulation of Mps1 at centrosomes. Mol Biol Cell. 2010;21(22): 3878–89. 10.1091/mbc.E10-04-0281 20861309 PMC2982088

[52] Kasbek C, Yang CH, Yusof AM, Chapman HM, Winey M, Fisk HA. Preventing the degradation of mps1 at centrosomes is sufficient to cause centrosome reduplication in human cells. Mol Biol Cell. 2007;18(11): 4457–69. 17804818 10.1091/mbc.E07-03-0283 PMC2043537

[53] Valenzuela-Muñoz V, Araya-Garay JM, Gallardo-Escárate C. SNP discovery and High Resolution Melting Analysis from massive transcriptome sequencing in the California red abalone *Haliotis rufescens* . Mar Genomics. 2013;10: 11–6. 10.1016/j.margen.2012.12.003 23353006

[54] Chen J, Chen ZS, Huang ZX, Ke CH, Zhang J, Zhong YX, et al Stable expression of Y-box protein 1 gene in early development of the abalone *Haliotis diversicolor* . Int J Dev Biol. 2012;56(5): 369–75. 10.1387/ijdb.113487zc 22689369

[55] Sakata K, Kashiwagi K, Igarashi K. Properties of a polyamine transporter regulated by antizyme. Biochem J. 2000;347(Pt 1): 297–303. 10727431 PMC1220960

[56] Ivanov IP, Gesteland RF, Atkins JF. A second mammalian antizyme: conservation of programmed ribosomal frameshifting. Genomics. 1998;52(2): 119–129. 9782076 10.1006/geno.1998.5434

[57] Zhu C, Lang DW, Coffino P. Antizyme2 is a negative regulator of ornithine decarboxylase and polyamine transport. J Biol Chem. 1999;274(37): 26425–30. 10473601 10.1074/jbc.274.37.26425

[58] He H, Kang B, Jiang D, Ma R, Bai L. Molecular cloning and mRNA expression analysis of ornithine decarboxylase antizyme 2 in ovarian follicles of the Sichuan white goose (*Anser cygnoides*). Gene. 2014;545(2): 247–52. 10.1016/j.gene.2014.05.022 24831833

[59] Ivanov IP, Rohrwasser A, Terreros DA, Gesteland RF, Atkins JF. Discovery of a spermatogenesis stage-specific ornithine decarboxylase antizyme: antizyme 3. Proc Natl Acad Sci USA. 2000;97(9): 4808–13. 10781085 10.1073/pnas.070055897 PMC18314

