# Supplementary figures and images for: Involvement of Antizyme Characterized from the Small Abalone Haliotis diversicolor in Gonadal Development
Source: PLoS One. 2015 Aug 27;10(8):e0135251. doi: 10.1371/journal.pone.0135251 (PMC4551804; doi:10.1371/journal.pone.0135251)

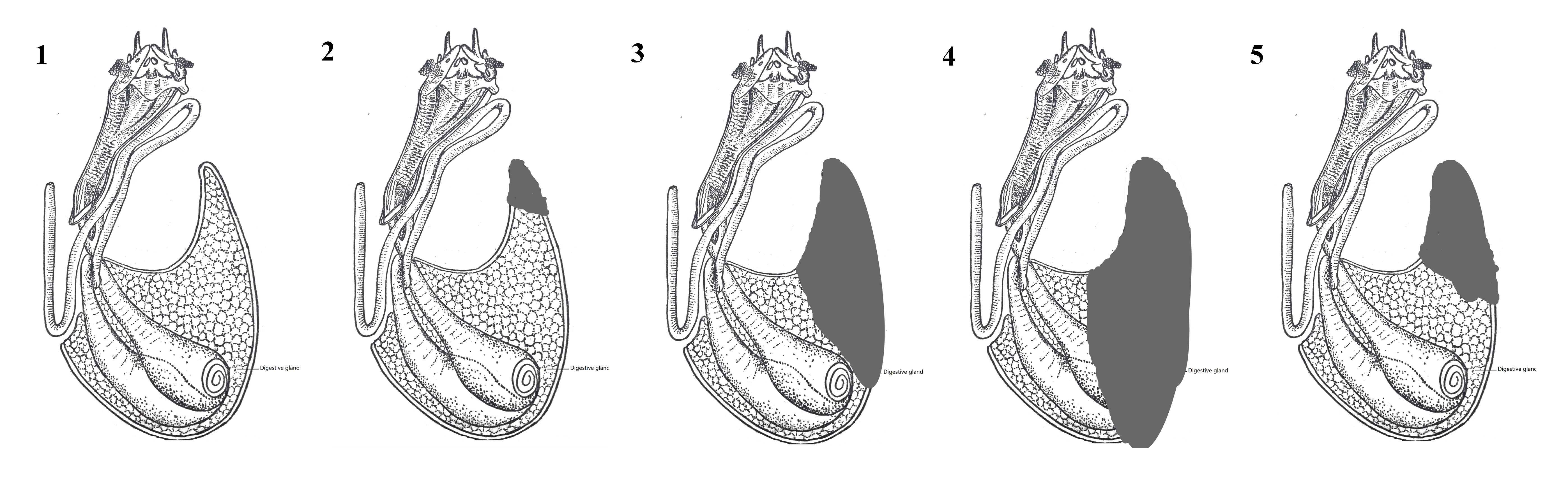

Supplement: S1 Fig — The digestive system of small abalone were shown. The gray area indicated the gonad tissues. The gonadal development were divided into five stages based on the size of gonad tissues or the extent of the covering on the digestive gland. (1) resting stage (2) proliferating stage (3) growth stage (4) maturing and spawning stage (5) final stage. (TIF) [file pone.0135251.s001.tif]
